# Roles of hepatic stellate cells in liver inflammation: a new perspective

**DOI:** 10.1186/s41232-016-0005-6

**Published:** 2016-04-25

**Authors:** Tomoko Fujita, Shuh Narumiya

**Affiliations:** Center for Innovation in Immunoregulatory Technology and Therapeutics, Faculty of Medicine, Kyoto University, Yoshida Konoecho, Sakyo-ku, Kyoto 606-8501 Japan

**Keywords:** Hepatic stellate cells, Liver sinusoids, Inflammatory cytokines, Hepatitis

## Abstract

Connected with the intestinal tract through the portal circulation, liver sinusoids function as the first line of defense against extrahepatic stimuli such as bacterial products and other toxic substances. Hepatic stellate cells (HSCs) are pericytes residing in the perisinusoidal space, between sinusoidal endothelial cells and hepatocytes, store vitamin A, and regulate sinusoidal circulation. Following chronic hepatitis, HSCs actively produce extracellular matrices and cause liver fibrosis. In spite of their close position to the liver sinusoids, however, whether HSCs contribute to liver inflammation has remained elusive. Evidence now accumulates to suggest that HSCs actively take part in the regulation of various forms of liver inflammation. Upon inflammatory stimuli from the sinusoids, HSCs produce various inflammatory molecules and interact with other liver cells, thereby recruiting and then activating infiltrating leukocytes and ultimately causing hepatocyte death. On the other hand, HSCs also exert hepatoprotective effects through inhibition of cytokine and chemokine production or induction of immunosuppressive cell population. HSCs therefore integrate cytokine-mediated inflammatory responses in the sinusoids and relay them to the liver parenchyma, either amplifying liver inflammation or suppressing parenchymal damage through immunoregulatory signaling depending on the context.

## Background

The liver is not only an organ of metabolism and detoxification but also the site where active immune responses take place. It is supplied by both systemic and portal circulation; 20 % of the blood comes from the hepatic artery and 80 % from the portal vein [1]. Various toxic substances, such as bacterial components and food antigens, are absorbed from the gut and first carried into the liver via the portal vein. Indeed, the concentrations of bacterial endotoxin in the systemic circulation were significantly lower than those of the portal vein [2], showing the potent filtering capacity of the liver.

Liver sinusoids, connected directly to the portal circulation, serve as the first barrier against these noxious stimuli. They contain various cell types, including sinusoidal endothelial cells, Kupffer cells (liver macrophages), and hepatic stellate cells (HSCs). Kupffer cells and sinusoidal endothelial cells, facing the sinusoidal lumen and in direct contact with the portal circulation, serve as the first line of defense against immune and inflammatory challenges [1, 3], producing inflammatory chemokines and cytokines and thereby attracting inflammatory cells from the systemic circulation and lymphoid organs.

HSCs reside in the space of Disse, the abluminal side of the sinusoids between liver sinusoidal endothelium and hepatocytes. They represent 5–8 % of all liver cells and 13 % of sinusoidal cells [4]. Physiological roles of HSCs include storage of vitamin A, synthesis of extracellular matrices (ECM) and matrix-degrading metalloproteinases, and regulation of sinusoidal blood flow. HSCs are regarded as pericyte equivalents in the liver. Like pericytes in other organs, HSCs are responsible for the regulation of blood flow and the production of ECM in inflammatory states. Upon liver injury, HSCs are activated, lose lipid-rich granules and transdifferentiate into α-smooth muscle actin (α-SMA)-positive myofibroblasts, which produce increased amount of ECM, and proinflammatory as well as profibrogenic cytokines, and cause liver fibrosis.

While the involvement of HSCs in liver fibrosis is well-recognized and attracts much attention [5], their role in liver inflammation has been little documented. Considering their anatomical position, HSCs appear to respond to inflammatory signaling from the sinusoids. HSCs from both human [6] and rodents [6, 7] produce cytokines and chemokines upon aberrant stimuli such as lipopolysaccharide (LPS) and other toxic substances, suggesting that HSCs can potentially regulate hepatic immune and inflammatory responses through their own gene expression. However, whether HSCs take part in the development of liver inflammation, and if so, whether they take on pro- or anti-inflammatory roles, is still controversial. We will review the recent findings on the roles of HSCs in acute and chronic liver inflammation.

## HSCs in immune-induced hepatitis

Concanavalin A (ConA) and LPS first activate immune cells in the sinusoids upon entry into the circulation and induce liver injury through massive production of inflammatory cytokines and chemokines by intra- and extrahepatic immune cells, followed by intraparenchymal infiltration of inflammatory cells [8]. Here, we discuss the roles of HSCs in these immune-mediated liver inflammation models.

### ConA-induced hepatitis

ConA is a lectin, a member of the family of carbohydrate-binding proteins. Intravenously injected ConA constitutively activates intrahepatic and systemic immune cells [9, 10]. Upon entry into the sinusoids, ConA binds to glycoproteins on the surface of Kupffer cells and sinusoidal endothelial cells. Sinusoidal endothelial cell barrier is disrupted by ConA [11, 12], which allows access of cytokines and sinusoidal cells to HSCs and hepatocytes. Inflammatory cytokines thus produced along with cells thus recruited in the liver cause massive liver injury. Among the numerous cytokines and chemokines produced in the liver, tumor necrosis factor (TNF)-α and interferon-γ (IFN-γ) are of high importance. Genetic deletion of TNF receptors [13] or IFN-γ [14] or neutralizing anti-TNF (TNF-α, lymphotoxin-α, and lymphotoxin-β) [15] or anti-IFN-γ antibody [16] attenuates ConA hepatitis in mice. In contrast to the above described roles of Kupffer cells and sinusoidal endothelial cells, contribution of HSCs to ConA hepatitis has not been investigated.

Our recent work [6] shows that HSCs receive inflammatory signals generated in the sinusoids and relay them to the liver parenchyma. In this work to clarify the role of prostaglandin D_2_ (PGD_2_) in liver pathophysiology, we found that mice deficient in PGD receptor DP1 showed exacerbated hepatitis after ConA injection, whereas administration of a DP1-specific agonist BW245C significantly suppressed liver inflammation induced by ConA. Bone marrow chimera studies suggested that DP1 expressed in non-hematopoietic cells is the target of BW245C. Consistently, DP1 is expressed exclusively in HSCs in the liver, while PGD synthase is induced in Kupffer cells after ConA injection, suggesting that PGD_2_ produced by Kupffer cells acts locally on HSCs to exert the protective effects. BW245C significantly suppressed intrahepatic and serum concentrations of TNF-α and IFN-γ, and DNA microarray analysis of the liver cDNA showed DP1 stimulation in HSC-inhibited expression of numerous TNF-α- and/or IFN-γ-inducible proinflammatory genes, which include *Nos2* (inducible nitric oxide synthase (iNOS)), *Tf* (tissue factor), *Edn1* (endothelin-1), *Vcam1* (vascular cell adhesion molecule-1 (VCAM-1)), and *Sele* (E-selectin). Decreased expression of these genes may have ameliorated hepatocyte damage, sinusoidal leukocyte accumulation, and hemostasis in ConA- and BW245C-treated mice.

In addition, our study also indicates that HSCs control CD4^+^ T cell trafficking to liver parenchyma. While CD4^+^ T cells were scattered in the parenchyma of the liver of ConA-treated mice, the cells failed to enter the liver parenchyma and formed clusters in periportal connective tissues in BW245C-administered, ConA-treated mice. Similar to skin pericytes which navigate neutrophils to the site of inflammation through intercellular adhesion molecule-1 (ICAM-1) [17], HSCs may serve as the hub for the intraparenchymal migration of CD4^+^ T cells. Since VCAM-1 is essential in CD4^+^ T cell adhesion on hepatic sinusoidal walls [18, 19], DP1-mediated suppression of lymphocyte migration from the periportal space to the liver parenchyma could in part be due to the decreased VCAM-1 expression by BW245C.

**Fig. 1:**
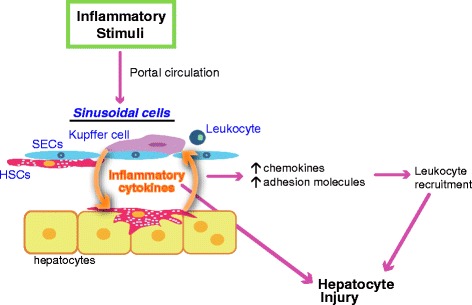
Schematic diagram of the proinflammatory effects of HSCs in liver inflammation. Inflammatory signals, such as bacterial products or toxic substances entering the liver through the portal circulation, first encounter immune cells and endothelial cells in the sinusoids. Immune cells (Kupffer cells and leukocytes) produce large amount of inflammatory cytokines and chemokines, which in turn damage endothelial cells and act on HSCs. Sinusoidal inflammation amplifies in a paracrine manner, causing hepatocyte injury through cytokines themselves or through infiltration of leukocytes into the liver parenchyma

These findings suggest the role of HSCs in ConA-induced hepatitis as follows. Upon ConA injection, massive production of inflammatory cytokines such as TNF-α and IFN-γ occurs in the sinusoids first by Kupffer cells and T cells and secondarily by HSCs in a paracrine manner. This amplification of cytokine production leads to a series of deleterious events (see Fig. 1), all of which contribute to massive liver injury. At the same time, PGD_2_ is produced by Kupffer cells, acts on DP1 of HSCs, and suppresses inflammatory responses to limit the amplification loop of liver inflammation. PGD_2_-DP1 system in the liver may thus serve as the brake on constitutive hepatic inflammation that would otherwise take place due to continuous external inflammatory stimuli carried into the liver via enterohepatic circulation.

### LPS/GalN-induced hepatitis

Intraperitoneal injection of LPS binds to TLR4 expressed in immune cells in the circulation and the sinusoids and activates them, which causes massive hemorrhagic liver injury through enhanced production of inflammatory cytokines and chemokines [20]. LPS is often injected simultaneously with a hepatotoxic agent d-galactosamine (GalN) to inhibit the protein synthesis and to promote cell death in hepatocytes, and this model of hepatitis is termed LPS and d-galactosamine (LPS/GalN) liver injury [20]. We found the suppression of LPS/GalN liver injury by DP1 stimulation and concomitant decrease in systemic and intrahepatic TNF-α levels [6]. Others observed attenuated liver injury after LPS administration in HSC-depleted mice, with decreased cytokine and chemokine expression [21]. These data, along with those of ConA-induced hepatitis, suggest that HSCs are involved in the pathogenesis of liver injury initiated by sinusoidal inflammation.

## HSCs in I/R liver injury

Ischemia and reperfusion (I/R) injury is a major complication of liver transplantation. It is induced by temporary liver ischemia of dissected liver graft and restoration of hepatic blood flow after transplantation. In liver transplantation, it is one of the major problems that affect the clinical outcomes [22]. During the ischemic period, hepatocytes are primarily affected by ischemia through mitochondrial damage and imbalances in pH and electrolytes. Endothelial cell damage, disturbance of microcirculation through up-regulation of a vasoconstrictive peptide endothelin-1 and its receptor endothelin-A receptor, and Kupffer cell activation also ensue. These lead to liver inflammation and injury through massive increase in reactive oxygen species and inflammatory mediators [22]. Reperfusion injury mainly involves reactive oxygen species generation by endothelial cells and neutrophils triggered by the reentry of oxygen into the liver tissue. During I/R liver injury, HSCs contribute to microcirculatory disturbances by acquiring a contractile phenotype through activation by inflammatory mediators [22].

Although HSCs have direct access to immune cells in the sinusoids due to endothelial cell damage, their role in the regulation of sinusoidal inflammation has not been studied in detail. However, several recent reports point to the proinflammatory properties of HSCs in I/R liver injury. A model for depleting HSCs was developed to elucidate the role of HSCs in I/R liver injury [21]. Transgenic mice expressing the herpes simplex virus thymidine kinase gene driven by the mouse GFAP promotor were treated with carbon tetrachloride to induce HSC proliferation and render them susceptible to cell killing induced by subsequent ganciclovir treatment. This method resulted in depletion of 64–72 % of HSCs. Liver injury caused by I/R, along with TNF-α, CXCL1, and endothelin-A receptor expression, was attenuated in HSC-depleted mice compared with controls. Histological studies further revealed decreased neutrophil infiltration and parenchymal cell death. These findings suggest that HSCs are, at least in part, involved in hepatic production of CXCL1 and contribute to neutrophil recruitment and microcirculatory failure caused by endothelin-A receptor stimulation.

Another group activated or depleted HSCs through pharmacological activation of endocannabinoid receptors CB1 or CB2, respectively [23], and examined their roles in I/R liver injury. CB1 stimulation activates HSCs and enhances liver fibrosis whereas that of CB2 causes HSC apoptosis [24], as evaluated by immunostaining for α-SMA. They first found that, after reperfusion, more than 25 % of CD4^+^ T cells adhering to the sinusoidal walls colocalized with HSCs in vehicle-treated mice. Activation of HSCs with a CB1 agonist increased non-perfused liver areas with no significant changes in CD4^+^ T cell recruitment, whereas their depletion with a CB2 agonist resulted in the attenuation of CD4^+^ T cell infiltration and reduced sinusoidal perfusion failure and liver enzyme activities. These data show that CD4^+^ T cells interact with HSCs before entering the hepatic parenchyma and suggest the possibility that HSCs may serve as sentinels in T cell migration into the parenchyma [23]. Hepatoprotective property of HSCs is also observed [25]. Adoptive transfer of HSCs induced the expansion of regulatory T cells (Treg cells) in the liver and provided partial protection against I/R liver injury, and depletion of Treg cells abolished the protective effect of HSCs, suggesting the cross talk between HSCs and Treg cells in hepatoprotection [25]. Further studies are required to define the precise roles of HSCs in I/R liver injury and delineate the spatiotemporal regulation of their actions.

## HSCs in NASH

Nonalcoholic steatohepatitis (NASH) is distinguished from nonalcoholic fatty liver in that NASH is characterized by the presence of intrahepatic inflammation accompanied by hepatocyte damage with or without fibrosis [26]. Upon liver injury, various liver resident cells such as Kupffer cells and HSCs are activated and, at the same time, inflammatory cells are recruited into the liver. This results in the amplification of intrahepatic inflammation and hepatocyte damage progresses further, leading to tissue fibrosis in some cases. Increased intestinal permeability is recently highlighted as a primary cause of liver injury in NASH [27]. Gut microbiota easily flow into the liver through the portal circulation, thus activating Toll-like receptors (TLRs) in liver cells, TLR4 in particular [28]. There have been numerous reports on the important roles of TLR4, which binds LPS. Serum LPS levels are elevated in patients with NASH and in animal models of NASH [29, 30], suggesting the activation of TLR4. TLR4 activation in HSCs induces the production of chemokines and the expression of adhesion molecules ICAM-1 and VCAM-1 [31, 32]. Furthermore, TLR4 signaling in HSCs promotes the interaction between HSCs and Kupffer cells by promoting the chemotaxis of Kupffer cells and up-regulating the expression of adhesion molecules in HSCs [32]. Down-regulation of transforming growth factor (TGF)-β pseudoreceptor BAMBI also occurs in HSCs by TLR4 ligation and activates TGF-β signaling, resulting in increased ECM production by HSCs and resulting fibrosis [32]. These findings provide evidence that HSCs are actively involved in the intrahepatic inflammation in NASH.

## HSCs in viral hepatitis

Hepatitis B and C virus (HBV and HCV, respectively) are main causes of chronic hepatitis and cirrhosis. In some patients, hepatocellular carcinoma (HCC) ensues, which in many cases is difficult to treat [33]. Hepatitis viruses replicate primarily in hepatocytes, causing mitochondrial injury and reactive oxygen species (ROS) formation [34]. Then, viral peptides presented with HLA elicit acquired immune responses by T cells [35]. Resident macrophages (Kupffer cells) and other immune cells recruited into the liver produce ROS and inflammatory as well as fibrogenic mediators. This results in the activation of HSCs accompanied by increased collagen synthesis [34].

Accumulating evidence suggests that HSCs play a role in HCV-induced chronic hepatitis [36]. The cross talk between HCV-infected hepatocytes and HSCs is demonstrated in an in vitro study [37]. Coculture of HSCs and HCV-infected hepatocytes induced CCL3 production by HCV-infected hepatocytes, which was mediated by HSC-derived IL-1α. IL-1α induces acute-phase response in the liver [38], and its expression is augmented in the liver of HCV-infected patients [39]. HCV proteins themselves can also elicit inflammatory and fibrogenic responses by HSCs [40]. HCV core protein induces cell proliferation and nonstructural proteins augment ICAM-1 expression and chemokine production through the NF-κB and c-Jun N-terminal kinase pathways in activated HSCs [40]. Finally, in the liver of chronic hepatitis C patients, CCR7 on HSCs induced cell migration and activation of several inflammatory pathways in response to CCL21 secreted by activated dendritic cells, resulting in induction of proinflammatory genes in HSCs [41].

## HSCs in HCC

HCC develops from several underlying diseases, such as viral hepatitis, alcoholic liver diseases, and NASH. The vast majority of HCC cases are preceded by liver fibrosis, with 90 % of hepatoma arising from cirrhotic livers [42]. Activated HSCs residing in the fibrous tissue, along with immune cells, contribute to the formation of tumor microenvironment favorable for tumor growth [43]. Tumor microenvironment is a mixture of tumor cells, stromal cells, and inflammatory molecules and ECM produced from the stromal cells, mainly HSCs in the case of HCC. Activated HSCs in the tumor stroma continuously produce ECM. Dysregulation of tissue inhibitor of metalloproteinases 1 (TIMP-1), which favors matrix deposition, and matrix metalloproteinases (MMPs), which degrade ECM, leads to increased collagen I deposition in the stroma and contributes to HCC progression [42, 44, 45]. HSCs in active state also produce soluble factors favoring tumor growth, such as hepatocyte growth factor and TGF-β [42], and proangiogenic factors such as vascular endothelial growth factor-A (VEGF-A) and MMP9 [46]. Malignant hepatocytes, in turn, produce inflammatory cytokines and chemokines that promote the survival of activated HSCs. HCC-HSC cross talk is vital in forming a tumor microenvironment that contributes to HCC survival and progression. In another concept, HSCs favor the survival and expansion of immunosuppressive cell populations. HSCs cause imbalance of T cell population by accelerating activated T cell apoptosis and expanding Treg cells [47–49]. Consistent with the above findings, HSCs cotransplanted with HCC cells support the implantation and growth of implanted tumor cells [47, 50].

HCC emerges from non-cirrhotic liver as well, notably in nonalcoholic fatty liver diseases including NASH [51]. Here, carcinogenesis has been linked to the secretion of inflammatory cytokines from adipose tissue, lipid accumulation in hepatocytes, and resulting hyperinsulinemia [51]. Unlike HCC resulting from chronic viral hepatitis, hepatoma cells in non-cirrhotic HCC are well-differentiated and tumors grow in a large nodular pattern [52]. It has been argued that HSCs may play only a minor role in this type of HCC, since larger liver nodules may be associated with diminished formation of fibrotic septa and therefore attenuation of HSC activation [51]. However, a recent study by Yoshimoto et al. associates prolonged activation of HSC and HCC development in obese mice without preceding cirrhosis [53]. They found that obesity alters gut microbiota and their metabolic product entered the liver through enterohepatic circulation, thus promoting hepatocarcinogenesis through sustained secretion of inflammatory mediators by HSCs. It should be noted that, in this model, chronic inflammation mainly contributes to hepatocarcinogenesis. This study shows the crucial role of HSCs as a bridge connecting intestinal flora and liver parenchyma in chronic liver inflammation.

## Conclusions

HSCs have traditionally been studied in the context of liver fibrosis. However, as reviewed here, recent studies show that they actively participate in liver inflammation by sensing external signals, producing inflammatory cytokines, and navigating T lymphocytes into the parenchyma (Fig. 1). In some context, HSCs can elicit anti-inflammatory actions as seen in DP1 stimulation in ConA-induced hepatitis and expansion of immunosuppressive cells in I/R liver injury and HCC. Further studies are required to elucidate the unexplored roles of HSCs in the development of hepatitis and clarify the factors that regulate different phenotypes of HSCs. As pericyte equivalents, further understanding of the physiology and inflammatory responses of HSCs will provide deeper insights into the actions of pericytes also in other organs and provide novel therapeutic targets in the treatment of inflammatory diseases of many organs including liver.

## Abbreviations

ConA: concanavalin A
ECM: extracellular matrix
HBV: hepatitis B virus
HCC: hepatocellular carcinoma
HCV: hepatitis C virus
HSCs: hepatic stellate cells
I/R: ischemia and reperfusion
ICAM-1: intercellular adhesion molecule-1
IFN-γ: interferon-γ
iNOS: inducible nitric oxide synthase
LPS: lipopolysaccharide
LPS/GalN: LPS and d-galactosamine
MMP: metalloproteinase
NASH: nonalcoholic steatohepatitis
PGD_2_: prostaglandin D_2_
ROS: reactive oxygen species
TGF-β: transforming growth factor-β
TIMP-1: tissue inhibitor of metalloproteinases-1
TLR: Toll-like receptor
TNF: tumor necrosis factor
Treg cells: regulatory T cells
VCAM-1: vascular cell adhesion molecule-1
VEGF-A: vascular endothelial growth factor-A
α-SMA: α-smooth muscle actin
